# Supplementary figures and images for: Pharmacological evaluation of mangrove plant Rhizophora mucronata (Lam.) grown in the coastal area of Sundarbans
Source: PLoS One. 2026 Jan 23;21(1):e0340646. doi: 10.1371/journal.pone.0340646 (PMC12829777; doi:10.1371/journal.pone.0340646)

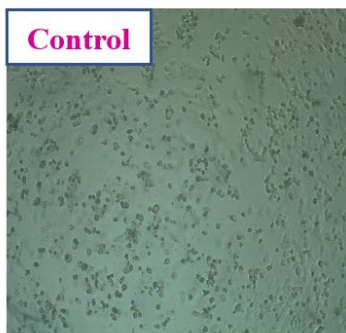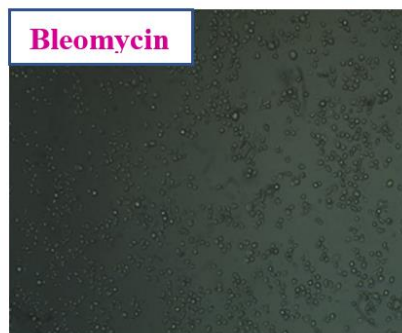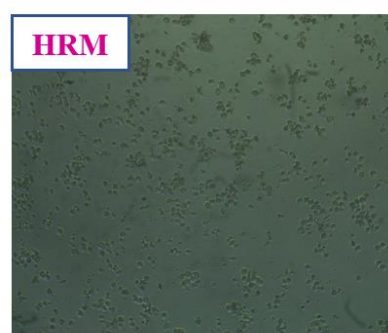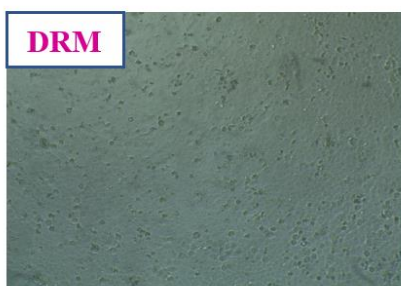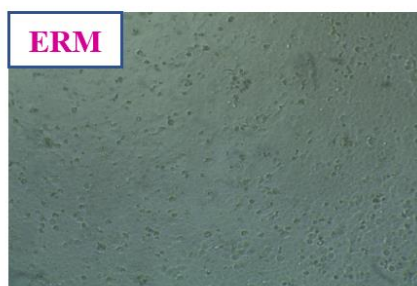

Supplement: S1 Fig — (PDF) [file pone.0340646.s001.pdf]

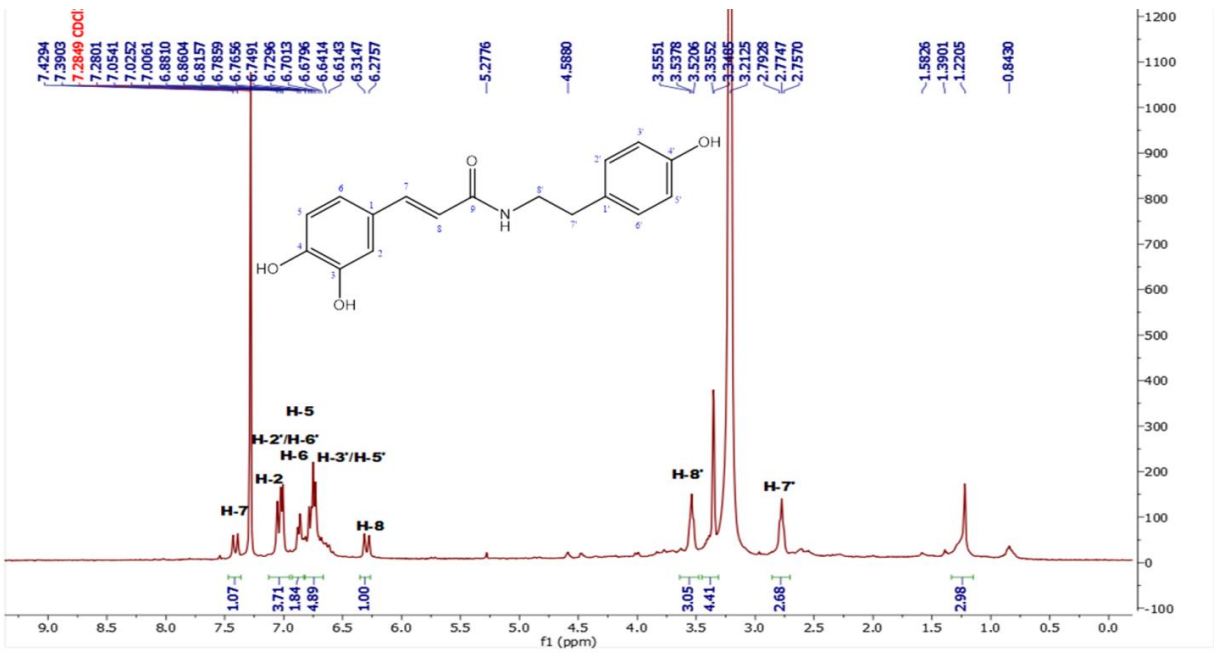

Supplement: S2 Fig — (PDF) [file pone.0340646.s002.pdf]

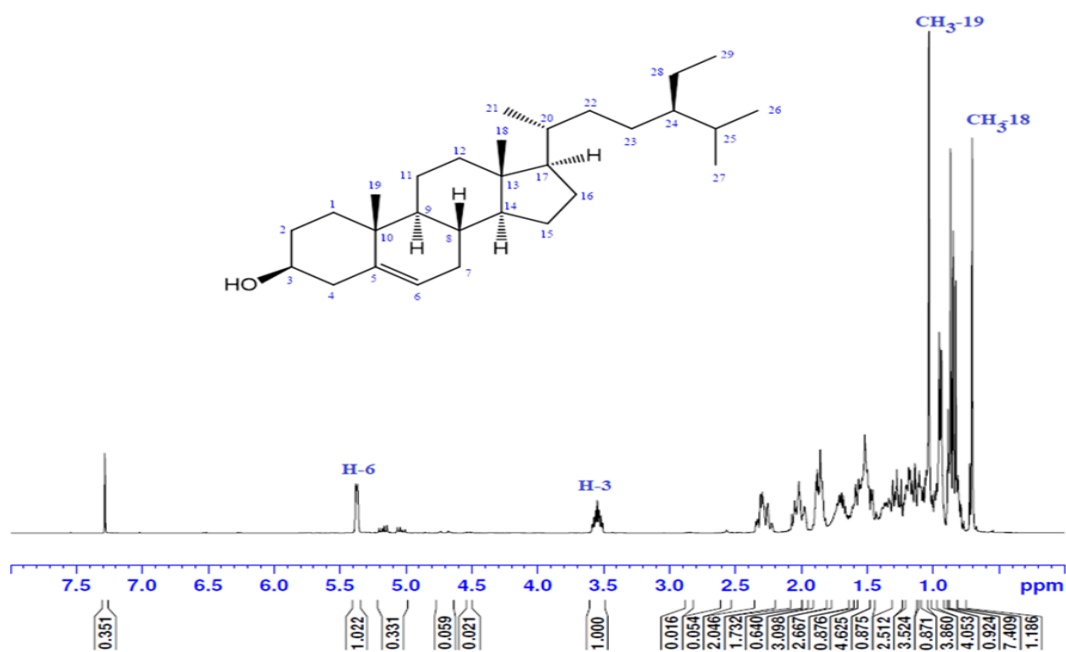

Supplement: S3 Fig — (PDF) [file pone.0340646.s003.pdf]
