# Supplementary material for: Pharmacological evaluation of mangrove plant Rhizophora mucronata (Lam.) grown in the coastal area of Sundarbans
Source: PLoS One. 2026 Jan 23;21(1):e0340646. doi: 10.1371/journal.pone.0340646 (PMC12829777; doi:10.1371/journal.pone.0340646)
Supplement: S4 Fig — (PDF) [file pone.0340646.s004.pdf]

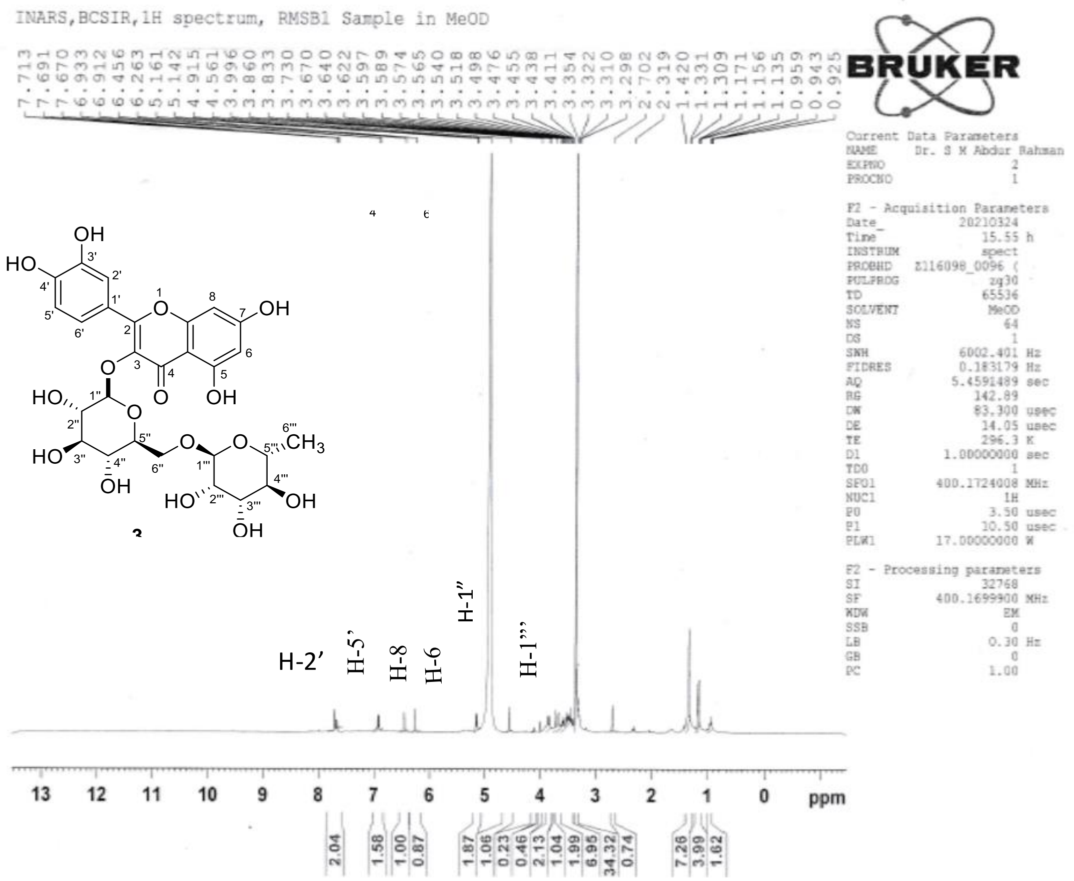
INARS,BCSIR,1H spectrum, RMSB1 Sample in MeOD
7.713 7.691 7.670 6.933 6.912 6.456 6.263 5.161 5.142 4.915 4.561 3.996 3.860 3.833 3.730 3.670 3.640 3.622 3.597 3.589 3.574 3.565 3.540 3.518 3.498 3.476 3.455 3.438 3.411 3.354 3.322 3.310 3.298 2.702 2.319 1.420 1.331 1.309 1.171 1.156 1.135 0.959 0.943 0.925
BRUKER
Current Data Parameters
NAME Dr. S M Abdur Rahman
EXPNO 2
PROCNO 1
F2 - Acquisition Parameters
Date_ 20210324
Time 15.55 h
INSTRUM spect
PULPROG zg30
TD 65536
SOLVENT MeOD
NS 64
DS 1
SWH 6002.401 Hz
FIDRES 0.183179 Hz
AQ 5.4591489 sec
RG 142.89
DW 83.300 usec
DE 14.05 usec
TE 296.3 K
D1 1.00000000 sec
TD0 1
SFO1 400.1724008 MHz
NUC1 1H
P0 3.50 usec
P1 10.50 usec
PLW1 17.00000000 W
F2 - Processing parameters
SI 32768
SF 400.1699900 MHz
WDW EM
SSB 0
LB 0.30 Hz
GB 0
PC 1.00
H-2'
H-5'
H-8
H-6
H-1"
H-1'''
3
ppm
13 12 11 10 9 8 7 6 5 4 3 2 1 0
2.04 1.58 1.00 0.87 1.87 1.06 0.23 0.46 2.13 1.04 1.99 6.95 34.32 0.74 7.26 3.99 1.62
