# Supplementary material for: Pharmacological evaluation of mangrove plant Rhizophora mucronata (Lam.) grown in the coastal area of Sundarbans
Source: PLoS One. 2026 Jan 23;21(1):e0340646. doi: 10.1371/journal.pone.0340646 (PMC12829777; doi:10.1371/journal.pone.0340646)
Supplement: S1 Table — (PDF) [file pone.0340646.s005.pdf]

Table S1. DPPH scavenging activity of RM fractions HRM, DRM and ERM and ascorbic acid.

Dose (µg/ml)	Ascorbic Acid	HRM	DRM	ERM
1	32.42±0.58	32.67±0.57	30.21±0.56	29.89±0.56
5	46.75± 0.59	35.21± 0.57	45.88± 0.52	37.33±0.34
10	54.67± 0.61	44.68±0.6	53.32± 0.301	51.47± 0.58
50	67.34± 0.59*	48.45± 0.6	57.87± 0.57	55.75± 0.57
100	81.32± 0.31*	51.22± 0.52	59.31± 0.56	76.14± 0.89*
500	86.44± 0.58*	71.21± 0.57*	82.23±0.87*	82.12± 0.57*

**Compound Code**	**R^2**	**IC_{50} (µg/mL)**
Ascorbic acid	0.9751	6.36
HRM	0.8985	36.59
DRM	0.9329	12.18
ERM	0.9347	11.7
