# Supplementary material for: Pharmacological evaluation of mangrove plant Rhizophora mucronata (Lam.) grown in the coastal area of Sundarbans
Source: PLoS One. 2026 Jan 23;21(1):e0340646. doi: 10.1371/journal.pone.0340646 (PMC12829777; doi:10.1371/journal.pone.0340646)
Supplement: S2 Table — (PDF) [file pone.0340646.s006.pdf]

Table S2: Antidiabetic effect of fractions HRM, DRM and ERM on streptozotocin induced diabetic mice.

Groups	Blood Glucose level (mmol/L)- Mean± SEM					
	**0 day**	**1st day**	**3rd day**	**5th day**	**7th day**	**% of lowering effect (7th Day)**
Group- I (Diabetic control)	20.9±4.4	18.3±2.7	16.3±4.3	20.1±3.2	21.8±5.2	----
Group-II	18.6±4.3	12.4±4.2	10.8±4.6	8.7±2.2	5.3±1.7*	71.5
Group-III	17.3±1.8	16.8±3.2	14.1±2.6	10.2±4.1	9.3±1.4*	49.1
Group-IV	19.1±2.2	15.7±2.8	12.2±1.9	9.1±1.7	7.5±2.8*	60.73
Group-V	18.4±1.9	15.5±2.5	11.4±3.8	10.1±2.1	8.8±1.6*	52.17

Values were expressed in Mean ± SEM. 0.5% Methyl cellulose was received by the diabetic control group and glibenclamide 5 mg/kg was received by reference group and HRM, DRM and ERM were given to Group-III, Group-IV and Group-V respectively at the dose of 200 mg/kg. *$p<0.05$ indicates significance compared with control group.
