# Supplementary material for: Pharmacological evaluation of mangrove plant Rhizophora mucronata (Lam.) grown in the coastal area of Sundarbans
Source: PLoS One. 2026 Jan 23;21(1):e0340646. doi: 10.1371/journal.pone.0340646 (PMC12829777; doi:10.1371/journal.pone.0340646)
Supplement: S3 Table — (PDF) [file pone.0340646.s007.pdf]

Table S3: *In-vitro* cytotoxic activity of HRM, DRM and ERM from RM on HeLa cell line.

Compound No.	Concentration (μg/ml))	% inhibition against HeLa cell line	IC_{50} value (μg/ml)
**Control (DMSO)**	**2.5**	**--**	**--**
**Bleomycin**	62.5	30.7±0.66	35.3
	125	70.4±.61	
	250	92.8±.64	
**HRM**	62.5	32.6±0.61	326.30
	125	45.5±0.65	
	250	52.8±0.89	
	500	68.9±0.55	
**DRM**	62.5	41.8±0.51	89.08
	125	54.3±0.52	
	250	70.5±0.60	
	500	76.8±0.45	
**ERM**	62.5	36.6± 0.63	127.6
	125	53.6± 0.79	
	250	68.4±0.55	
	500	74.2±0. 89	

Note: DMSO = Dimethyl sulfoxide
