# Supplementary material for: Pharmacological evaluation of mangrove plant Rhizophora mucronata (Lam.) grown in the coastal area of Sundarbans
Source: PLoS One. 2026 Jan 23;21(1):e0340646. doi: 10.1371/journal.pone.0340646 (PMC12829777; doi:10.1371/journal.pone.0340646)
Supplement: Attachment — Submitted filename: Review 2025.docx [file pone.0340646.s008.docx]

**Review on manuscript PONE-D-25-31376**

In this paper, the authors report on the investigation of the anti-diabetic, antibacterial, anti-cancer and anti-oxidant properties of hexane, dichloromethane and ethylacetate extracts of the leaves of the mangrove R. Mucronata. In depth in vitro and in vivo studies were conducted. Three compounds were isolated from the extracts and successfully characterised by NMR.

Overall, the research methodology is sound. However, the authors failed to highlight previous studies related to the current one and they did not clearly point out the novelty of the current investigation. Therefore, the manuscript requires major revision before it can be considered for publication.

**Major comments:**

1. The abstract should be reformulated and better presented to readers to have a better flow and understanding of the study rationale and what investigations have been carried out highlighting key results only.
2. The introduction needs to be further consolidated with scientific reports on the biological properties of this mangrove extracts. The authors should highlight how the current study is novel and adds to already reported literature. Consider publications such as Sadeer et al., 2019

Moreover, there are recent US patents on drug formulations derived from extracts of R. muncrata.

It is not clear why the authors chose only the leaf of the plant and did not consider other parts of the plant.

3. Regarding the in vivo anti-cancer study, can the authors provide a reference to support the protocol that was used.
4. The results should be discussed and compared with already reported data.
5. Consider depicting the results in the tables in the form of figures instead.
6. The figures are of poor quality.
7. Please conduct statistical analysis on figures 1 and 3.
8. Provide the 1H-NMR spectra of the three isolated compounds and assign the peaks to the structure.
9. Consolidate the discussion by linking the anti-cancer, anti-diabetic, anti-oxidant properties etc to the functional groups in the isolated compounds.

**Minor comments:**

1. Define the abbreviations used in the abstract
