# Supplementary material for: Pharmacological evaluation of mangrove plant Rhizophora mucronata (Lam.) grown in the coastal area of Sundarbans
Source: PLoS One. 2026 Jan 23;21(1):e0340646. doi: 10.1371/journal.pone.0340646 (PMC12829777; doi:10.1371/journal.pone.0340646)
Supplement: Attachment — Submitted filename: Response to Reviewers.docx [file pone.0340646.s010.docx]

13.12.2025

Dear Editor:

Thanks to respected editor and the reviewers for your nice and constructive comments. According to the editorial and reviewer’s suggestions, we have revised the manuscript and are submitting a modified version of our manuscript. The revision is marked with a yellow color in the manuscript.

**Editorial Response:**

Major comments:

1.      Please ensure that your manuscript meets PLOS ONE's style requirements, including those for file naming.

**Author’s reply:** Thank you for the generous suggestions. The manuscript has been revised accordingly. We have added the authors name, title and affiliations in the front page.

2.      In your Methods section, please provide additional information regarding the permits you obtained for the work. Please ensure you have included the full name of the authority that approved the field site access and, if no permits were required, a brief statement explaining why.

**Author’s reply:** Thank you. Usually collecting the common species from the nearby areas of sundarban’s does not require any official permission. However, we had taken a verbal permission from the local forest office which has been mentioned in the manuscript as follows:

Fresh leaves of the plant *Rhizophora mucronata* weredss collected from mangrove Sundarbans Bangladesh in February 2019 upon taking verbal permission from the local forest officer at Karamjal, Sundarban area without disturbing its inhabitant properties. (section 2.2)

3.      To comply with PLOS ONE submissions requirements, in your Methods section, please provide additional information regarding the experiments involving animals and ensure you have included details on (1) methods of sacrifice, and (2) efforts to alleviate suffering.

**Author’s reply:** We have revised the manuscript and added a section (section 2.6) for handling animals. According to your suggestion, we have further included detail animal handling and sacrifice methods. The following section (section 2.6) has been included in the manuscript:

**2.6 Animals**

Healthy adult locally bred Swiss albino mice, aged 4 weeks (body weight ranges from 30-40 gm) were collected from the North South University, Bangladesh. Adequate supply of food and water were ensured and kept them into a 12h darkness-light and ambient room temperature (25±2 ^0^C) for adopting the laboratory environment. Before oral delivery of any test samples the mice were kept into fasting conditions for at least 12-hour prior of experiment. All the animal related procedures were carried out following the institutional animal care handling protocol which was approved by ethical committee of Faculty of Biological Sciences, University of Dhaka (Approval. number. 187/Biol. Sc.; December 20, 2022). Moreover, precautions were strictly taken to adhere with International Council for Laboratory Animal Science, the Nuffield Council on Bioethics (NCB), and the Council for International Organization of Medical Sciences (CIOMS/ICLAS) guidelines. All efforts were ensured to alleviate their sufferings. Finally. mice were euthanized by injecting phenobarbital sodium (125 mg/kg body weight) intraperitoneally. Death of the mice was confirmed by cessation of the chest movement and no response to toe pinch and finally the dead mice were disposed according to the institutional guidelines.

4.      Please ensure that you include a title page within your main document. We do appreciate that you have a title page document uploaded as a separate file, however, as per our author guidelines (<http://journals.plos.org/plosone/s/submission-guidelines#loc-title-page>) we do require this to be part of the manuscript file itself and not uploaded separately.

**Author’s reply:** Thank you. We have revised the manuscript accordingly.

5.      Please include captions for your Supporting Information files at the end of your manuscript, and update any in-text citations to match accordingly. Please see our Supporting Information guidelines for more information: <http://journals.plos.org/plosone/s/supporting-information>

**Author’s reply:** Captions for supporting information have been added at the end of the manuscript

6.      If the reviewer comments include a recommendation to cite specific previously published works, please review and evaluate these publications to determine whether they are relevant and should be cited. There is no requirement to cite these works unless the editor has indicated otherwise.

**Author’s reply:** This suggestion has been followed and no unnecessary reference has been included.

**Reviewer 1 Responses:**

1.      Correct comma usage in English language - in abstract.

**Author’s reply:** Thank you for the suggestion. The abstract has been revised accordingly.

2.      Item 2.2 Plant materials - also correct comma usage in English language.

**Author’s reply:** The comma usage in English language is corrected accordingly.

3.      Item 2.4 General phytochemical screening: Preliminary phytochemical analysis was performed to understand the types of constituents present according to the standard methods [could you describe more about it?].

**Author’s reply:** Thank you for the suggestion. The following sentences has been mentioned in the manuscript:

Presence of various plant secondary metabolites such as alkaloids, tannins, flavonoids, steroids, phenols, saponins, terpenoids, glycosides and quinones are screened using different standard chemical tests. The description of all the chemical tests was also included in section 2.4.

4.      Item 2.5: "The % inhibition or percentage of DPPH scavenging was estimated by applying the following equation" [I suggest use of "percentage" - not both symbol and word].

**Author’s reply:** Thank you. This has been revised accordingly (Section 2.5 and 2.11).

5.      Figure: for me the figures look blurry, needing more resolution.

**Author’s reply:** Thank you. We have improved the resolution of the figures.

**Reviewer 2 Responses:**

In this paper, the authors report on the investigation of the anti-diabetic, antibacterial, anti-cancer and anti-oxidant properties of hexane, dichloromethane and ethylacetate extracts of the leaves of the mangrove R. Mucronata. In depth in vitro and in vivo studies were conducted. Three compounds were isolated from the extracts and successfully characterised by NMR.

Overall, the research methodology is sound. However, the authors failed to highlight previous studies related to the current one and they did not clearly point out the novelty of the current investigation. Therefore, the manuscript requires major revision before it can be considered for publication.

1. The abstract should be reformulated and better presented to readers to have a better flow and understanding of the study rationale and what investigations have been carried out highlighting key results only.

**Author’s reply:** Thank you for the insightful suggestion. Abstract has been revised accordingly as shown below:.

*Rhizophora mucronata*, a mangrove species native to coastal region of Bangladesh, has been drawn significant interests due to its potential ecological and therapeutic values, particularly as antioxidants, antibacterial and anti-inflammatory properties. The current investigation was aimed to identify the bioactive compounds from the different fractions of *Rhizophora mucronata* and to determine their antioxidants, analgesic, hypoglycemic, antimicrobial along with *in-vitro* and *in-vivo* anticancer properties. Total three known compounds namely *N*-trans-para-caffeoyl-tyramine **(1)***,* β-sitosterol **(2)** and rutin **(3)** were isolated from the ethyl acetate fractions (ERM) and their structures were elucidated by analyzing ^1^H-NMR spectral data. Dichloromethane (DRM) and ethyl acetate (ERM) fractions showed significant free radical scavenging properties (IC_50_ value 12.18 and 11.7 μg/mL, respectively) compared to the standard ascorbic acid (6.36 μg/mL) in DPPH free radical scavenging assay. All three fractions exhibited notable analgesic effect in mice compared to standard drug diclofenac sodium in acetic acid induced writhing method. DRM and ERM fractions revealed significant glucose lowering effects compared to standard glibenclamide in streptozotocin induce diabetic mice model. Besides, all the fractions showed remarkable antibacterial effects (zone of inhibition 11.1-17.3 mm) against all selected *Gram-positive* but showed moderate activity against the *Gram-negative* bacteria. *In-vitro* cytotoxicity test of DRM and ERM fractions exhibited cytotoxic effect (IC_50_ value 88.94 µg/ml and 127.6 µg/ml, respectively) against HeLa cell. The *in-vivo* cell growth inhibition of the three fractions on EAC (Ehrlich ascites carcinoma) cell demonstrated that ERM fraction furnished maximum cell growth inhibition (54.61%) compared to 84.83% inhibition by bleomycin.*,*. From the above findings, it is evident that ethyl acetate fractions of *R. mucronata* can be exploited for future drug development and traditional medicinal applications.

2. The introduction needs to be further consolidated with scientific reports on the biological properties of this mangrove extracts. The authors should highlight how the current study is novel and adds to already reported literature. Consider publications such as Sadeer et al., 2019

Moreover, there are recent US patents on drug formulations derived from extracts of R. Mucronata. It is not clear why the authors chose only the leaf of the plant and did not consider other parts of the plant.

**Author’s reply:** Revised accordingly. Following texts have been added to introduction section in the manuscript.

Crude methanolic extract of *Rhizophora mucronata* has been reported to have anticancer properties in different cancer cell lines [9]. 80% methanolic extract of R. mucronata leaves possesses flavonoids, gallic acid, quercetin and coumarin that has antiradical, antihyperglycemic and reduced lipid peroxidation activities. [10]. Detailed assessment of the traditional uses such as anticancer, anti-inflammation, antimicrobial, antidiabetic etc along with 60 chemical components and their toxicity studies from R. mucronata has been reported earlier [11]. The antidiarrheal and anti-inflammatory activity guided isolation of bioactive components has led to isolate six compounds from R. mucronata bark extracts. [12] Tannins such as catechin, epigallocatechins were reported from the leaf extract of R. mucronata that relaxed or reduced the contractions of ileum in diarrheal rats. [13]

**Please see also authors reply of query no 11**

The leaf of the plant was chosen because of the following reason which has been included in the introduction section:

Leaf extracts of *R. mucronata* were chosen because very little investigation in the pharmacological activity of leaf extracts have been reported. Moreover, studies indicated that the high concentration fruit flour of *R. mucronata* contains trace amounts of hydrogen cyanide (HCN) leading to create toxicity [15]. Alternatively, fresh leave extract of *R.* *mucronata* is considered safe and possessed no hazardous effect at standard therapeutic concentration [16]

3. Regarding the in vivo anti-cancer study, can the authors provide a reference to support the protocol that was used.

**Author’s reply:** Thank you for your suggestion. The following write up has been mentioned in the manuscript along with referenced protocol:

In-vivo cell growth inhibition was carried out following the established protocol as described earlier [27]. Five groups of Swiss albino mice (n=5) were used for this study. (section 2.11)

4. The results should be discussed and compared with already reported data.

**Author’s reply:** Thank you for the suggestion. The results are compared with other data and the manuscript was revised accordingly. (Section 4; Discussion).

In case of antioxidant and antimicrobial properties, the results were already compared with the reported data (Section 4; discussion). In addition, the following sentences were added to compare the results of the antidiabetic, analgesic and anticancer effects:

In an earlier investigation, ethanolic extracts of *R. mucronata* showed 44% reduction in the writhing with a dose of 200 mg/kg body weight in mice which is in accordance with our findings [35]. The current extracts from Sundarbans showed better writhing inhibition.

Our findings reestablish the previous findings where ethanolic fractions of *R. mucronata* significantly inhibited α-amylase and α-glucosidase enzymes activity as well as controlled glycemic index and reduced diabetic complications [37].

These results reinforce the findings of a previous in vitro anticancer study of R. mucronate crude methanol extracts where the extracts were found to be effective against colon, lung, prostrate and breast cancers [9].

5. Consider depicting the results in the tables in the form of figures instead.

**Author’s reply:** Thank you. Some tables and figures have been changed ( previous **Table 2** was transformed to **Fig 2**).

6. The figures are of poor quality.

**Author’s reply:** The quality and resolution of figures are improved accordingly.

7. Please conduct statistical analysis on figures 1 and 3.

**Author’s reply:** Statistical analysis was accomplished and mentioned in the text.

8. Provide the 1H-NMR spectra of the three isolated compounds and assign the peaks to the structure

**Author’s reply:** Thank you for the suggestion. We have provided NMR spectra in the supporting files and assigned the peaks as well. The complete spectral data is present in the manuscript.

9. Consolidate the discussion by linking the anti-cancer, anti-diabetic, anti-oxidant properties etc to the functional groups in the isolated compounds

**Author’s reply:** We have tried our best to incorporate few points on it. For example, in case of antioxidant and antimicrobial activity we have already included the following sentence:

Therefore, the highest activity of the comparatively polar ERM fraction indicates that polar compounds might be responsible for antioxidant effect. Plants are rich sources of flavonoids and phenolic compounds which have the ability to scavenge free radicals due to their ability to donate hydrogen atom [34]. Phytochemical investigation of ERM fraction provided phenolic compound *N*-trans-para-caffeoyl-tyramine (**1**) and flavonoids rutin (**3**) which might have contributed for the strong antioxidant property of ERM fraction*.*

The potent antimicrobial activity of the crude extracts may be due to the presence of phenols, flavonoids, alkaloids, steroids [43].

10. Define the abbreviations used in the abstract

**Author’s reply:** Abbreviations have been introduced accordingly.

11. They did not clearly point out the novelty of the current investigation.

**Author’s reply:** Novelty and Rational of the current investigation is described in Introduction and Conclusion section. The following sentences were added in introduction section which describe some novel approach for phytochemical and biological activity:

Although enormous investigation on *R. Mocronata* whole plant extracts has been accomplished previously, only few reports are found on different parts of the plant. Moreover, biological investigation on differential extracts or fractions depending on polarity of the solvents are missing. Moreover, comprehensive pharmacological screening including in vitro and in vivo anticancer studies along with detailed understanding and characterization of bioactive components are very rare. Leaf extracts of *R. mucronata* were chosen because very little investigation in the pharmacological activity of leaf extracts have been reported. (Section 1: Introduction)

The current study has been designed to investigate various pharmacological activities such as antioxidants, analgesic and anti-inflammatory, antidiabetic, antimicrobial and cytotoxic potentials with different fractions (HRM, DRM and ERM) of *R. mucronata*. Moreover, in this research we focused on isolation and characterization of active constituents from the active fraction of *R. mucronata*

Here three compounds *N*-trans-para-caffeoyl-tyramine (**1**) and β-sitosterol (**2**) and rutin (**3**) from ERM fraction of *R. mucronata* and *N*-trans-para-caffeoyl-tyramine (**1**) and rutin (**3**) were isolated from this plant for the first time. The fractions also gave significant *in-vitro* cytotoxic effect on HeLa cell and *in-vivo* anticancer effect against EAC cell which were performed for the first time by this plant extracts. (Section 5: Conclusion)

12. It is not clear why the authors chose only the leaf of the plant and did not consider other parts of the plant.

**Author’s reply:** The leaf of the plant was chosen because of the following reason which has been included in the introduction section:

Although enormous investigation on *R. Mocronata* whole plant extracts has been accomplished previously, only few reports are found on different parts of the plant. Moreover, biological investigation on differential extracts or fractions depending on polarity of the solvents are missing. Leaf extracts of *R. mucronata* were chosen because very little investigation in the pharmacological activity of leaf extracts have been reported. Moreover, studies indicated that the high concentration fruit flour of R. mucronata contains trace amounts of hydrogen cyanide (HCN) leading to create toxicity [15]. Alternatively, fresh leave extract of *R. mucronata* is considered safe and possessed no hazardous effect at standard therapeutic concentration [16].
